# Comparison of Two Central Venous Pressure Control Strategies to Prevent Atrial Fibrillation After Coronary Artery Bypass Grafting

**DOI:** 10.5935/abc.20170044

**Published:** 2017-04

**Authors:** Mario Augusto Cray da Costa, Wesley Lirani, Ana Caroline Wippich, Luana Lopes, Eduardo de Souza Tolentino, Beatriz Zampar, Marcelo Derbli Schafranski

**Affiliations:** Universidade Estadual de Ponta Grossa, Ponta Grossa, PR - Brazil

**Keywords:** Central Venous Pressure, Atrial Fibrillation/prevention, Myocardial Revascularization, Postoperative Care

## Abstract

**Background:**

Atrial fibrillation (AF) takes place in 10-40% of patients undergoing coronary artery bypass grafting (CABG), and increases cardiovascular mortality. Enlargement of atrial chambers is associated with increased AF incidence, so patients with higher central venous pressure (CVP) are expected to have larger atrial distension, which increases AF incidence.

**Objective:**

To compare post-CABG AF incidence, following two CVP control strategies.

**Methods:**

Interventional, randomized, controlled clinical study. The sample comprised 140 patients undergoing CABG between 2011 and 2015. They were randomized into two groups, G15 and G20, with CVP maintained ≤ 15 cmH_2_O and ≤ 20 cmH_2_O, respectively.

**Results:**

70 patients were included in each group. The AF incidence in G15 was 8.57%, and in G20, 22.86%, with absolute risk reduction of 14.28%, and number needed to treat (NNT) of 7 (p = 0.03). Mortality (G15 = 5.71%; G20 = 11.42%; p = 0.07), hospital length of stay (G15 = 7.14 days; G20 = 8.21 days; p = 0.36), number of grafts (median: G15 = 3, G2 = 2; p = 0.22) and cardiopulmonary bypass use (G15 = 67.10%; G20 = 55.70%; p = 0.22) were statistically similar. Age (p = 0.04) and hospital length of stay (p = 0.001) were significantly higher in patients who developed AF in both groups.

**Conclusion:**

Keeping CVP low in the first 72 post-CABG hours reduces the relative risk of AF, and may be useful to prevent AF after CABG.

## Introduction

Atrial fibrillation (AF) is an arrhythmia that results from abnormal depolarization, causing loss of the atrial contraction ability. It is related to increased risk for stroke and mortality.^1-3^

Atrial fibrillation in the postoperative period (PO) of coronary artery bypass grafting (CABG) occurs in 5-40% of patients, usually from the second to the fourth PO day, peaking on the second day.^3,4^ Its pathophysiology is multifactorial and includes oxidative stress, systemic inflammatory response, excessive production of catecholamines, changes in autonomic tone and connexin expression. Such factors cause dispersion of atrial refractoriness, alter atrial electrical conduction and predispose to arrhythmia.^4,5^

The following risk factors are related to the higher incidence of post-CABG AF: advanced age, peripheral vascular disease, chronic obstructive pulmonary disease (COPD), diabetes mellitus, systemic arterial hypertension, valvular heart disease, left atrial enlargement, left ventricular dysfunction, history of previous AF or acute myocardial infarction (AMI), suspension of beta-blockers in the preoperative period, use of cardiopulmonary bypass (CPB), and increased PO sympathetic tone.^6,7^

Atrial fibrillation in the PO period of CABG worsens the patient's hemodynamic status, because of the increased risk for congestive heart failure and embolic events in the long run. Stroke is a major complication, observed in 2% of surgical patients. In addition, AF has been associated with higher in-hospital mortality and worse survival in the long run.^4-7^ Such complications justify the need for prophylactic measures for post-CABG AF.

The rationale of this study is based on the following hypothesis: the increased volume of the atria is associated with increased AF incidence;^8^ therefore, patients with higher central venous pressure (CVP) are expected to have greater atrial distension, and, thus, a higher AF incidence, in addition to being predisposed to pulmonary congestion, hypoxemia and atrial wall edema, factors that contribute to increase the incidence of that arrhythmia. Thus, CVP control could be useful to prevent post-CABG AF. Aiming at testing that hypothesis, the post-CABG AF incidence was assessed under two CVP control strategies.

## Objective

This study aimed at assessing whether the incidence of AF 48 to 72 hours after CABG differs between two CVP control strategies (based on intent to treat), by comparing two groups: G15 (CVP ≤ 15 cmH_2_O) and G20 (CVP ≤ 20 cmH_2_O), AF being the primary outcome. The secondary outcome was to compare hospital length of stay after CABG, as well as mortality, between both groups.

## Methods

### Type of study

This is a clinical, randomized, controlled, interventional and prospective study performed at the Intensive Care Unit (ICU) of Santa Casa de Misericórdia of Ponta Grossa, Paraná State, in partnership with the State University of Ponta Grossa (UEPG). The analysis was performed with data prospectively collected. This study project was approved by the Ethics Committee in Research UEPG, abides by the 1975 Declaration of Helsinki, and all participants provided written informed consent before surgery.

### Inclusion and exclusion criteria

This study sample comprised patients undergoing CABG at the Cardiac Surgery Service of the Santa Casa de Misericórdia of Ponta Grossa from January 2011 to December 2015.

Patients with the following characteristics were excluded: undergoing CABG in association with another procedure; history of preoperative AF; contraindication to maintain CVP below the established values (such as severe pulmonary hypertension); severe chronic renal failure, determined by a glomerular filtration rate < 30 mL/min; severe left ventricular dysfunction; use of high doses of vasoactive drugs, such as dopamine or dobutamine > 7mcg/kg/min or noradrenaline > 0.7mcg/kg/min; no use of beta-blocker or statin in the preoperative period, or no diet reintroduction, and after vasoactive drug suspension in the PO period; need for more than 20 ampoules of furosemide within 24 hours to maintain CVP levels.

### Outcomes

**Primary outcome:** presence of AF 48 to 72 hours after CABG, on continuous electrocardiographic monitoring (cardioscope) and documented on 12-lead electrocardiogram (ECG).

**Secondary outcomes:** in-hospital mortality and hospital length of stay after CABG.

### Definition of the groups and data collection

Patients were randomized into two groups by use of draw: G15, with a CVP goal of ≤ 15 cmH_2_O; and G20, with a CVP goal of ≤ 20 cmH_2_O. The strategy of CVP control consisted of measuring CVP every 2 hours for 72 hours after CABG, or until discharge from the ICU. The minimum ICU length of stay was 48 hours. Whenever CVP reached its cutoff point, a furosemide ampoule was intravenously administered, from the sixth hour on, because, in the first 6 post-CABG hours, hemodynamic instability is higher. Vasoactive or anti-hypertensive drugs were administered to maintain a mean arterial pressure (MAP) of 60-100 mm Hg. The AF incidence was compared between the groups, detected by use of continuous electrocardiographic monitoring (cardioscope) and confirmed on 12-lead ECG.

The following data were collected daily: CVP levels, need for furosemide, AF occurrence within the post-CABG period (48 to 72 hours), hospital length of stay and in-hospital death.^9^ The following surgical data and comorbidities were collected from the patients' standard preoperative and PO forms: age, sex, previous AF, diabetes mellitus, COPD, chronic kidney disease, peripheral vascular disease, left ventricular function, recent AMI, use of CPB, and number of grafts.

### Statistical analysis

The statistical analysis was performed in two steps. In the first, the following variables were compared between the G15 and G20 groups: age, sex, diabetes mellitus, COPD, peripheral arterial disease, recent AMI within three months from surgery, moderate left ventricular dysfunction (ejection fraction < 50% and > 35%), glomerular filtration rate, AF incidence, in-hospital death, post-CABG hospital length of stay (days), number of grafts, and use of CPB. The quantitative variables were expressed as medians for nonparametric data, or as means for parametric data, and coefficient of variation (CV). The qualitative variables were expressed as absolute numbers and percentages. In the second step, the patients were divided into two groups, one group of those who developed AF and the other group of those who did not developed AF, and the following variables were compared between the new groups: age, in-hospital death, post-CABG hospital length of stay (days), number of grafts, and use of CPB. Their statistical analysis was performed with the MedCalc software. The qualitative variables were assessed by using two-tailed Fisher exact test. The quantitative variables had their normality tested by use of Shapiro-Wilk test; because the data had nonparametric distribution, they were assessed by use of two-tailed Mann-Whitney test. To assess the effect size, absolute risk reduction (ARR) and relative risk reduction (RRR) were used, and, for qualitative variables, the number needed to treat (NNT). The statistical significance level adopted was p < 0.05.

## Results

The sample comprised 140 patients randomized into two groups, with 70 patients each: G15, CVP maintained ≤ 15 cmH_2_O; and G20, CVP maintained ≤ 20 cmH_2_O. The comparative analysis of patients' age and sex is shown in Table 1. The mean ages of the groups were 60 years (CV = 0.17) in G15, and 63 years (CV = 0.15) in G20 (p = 0.6). The male sex predominated in both groups (G15=67.10%; and G20 = 81.43%; p = 0.07).

**Table 1:** Patients' age and sex according to group (G15: CVP control ≤ 15 cmH_2_O; and G20: CVP control ≤ 20 cmH_2_O)

Variables	G15 (n = 70)	G20 (n = 70)	p
Age, mean (CV)	60 (0.17)	63 (0.15)	0.6 ^†^
Male sex, n (%)	47 (67.1)	57 (81.43)	0.07*

(*) Fisher exact test (two-tailed);

(†) Mann-Whitney test (two-tailed); CVP: central venous pressure; CV: coefficient of variation.

The analysis of comorbidities evidenced no statistical difference between the groups (Table 2).

**Table 2:** Assessed comorbidities according to group (G15: CVP control ≤ 15 cmH_2_O; and G20: CVP control ≤ 20 cmH_2_O)

Variables	G15 (n = 70)	G20 (n = 70)	p
Diabetes mellitus, n (%)	18 (25.71)	25 (35.71)	0.07*
COPD, n (%)	10 (14.28)	11 (15.71)	1.00*
Peripheral arterial disease, n (%)	7 (10.00)	12 (17.14)	0.27*
Previous recent AMI, n (%)	28 (40)	23 (32.85)	0.48*
LVD (EF < 50%), n (%)	8 (11.42)	16 (22.85)	0.11*
Renal function (GFR), mL/min (CV)	85.78 (0.37)	88.32 (0.49)	0.48 ^†^

(*) Fisher exact test (two-tailed);

(†) Mann-Whitney test (two-tailed); CVP: central venous pressure; COPD: chronic obstructive pulmonary disease; AMI: acute myocardial infarction; LVD: left ventricular dysfunction; EF: ejection fraction; CV: coefficient of variation; GFR: glomerular filtration rate.

In the 48-72 post-CABG hours, the incidence of AF differed statistically between the groups (Table 3). In G15, 8.56% of the patients developed AF, in contrast to 22.86% of those in G20 (p = 0.03). The ARR was 14.28% [95% confidence interval (95% CI): 2.14-26.28], and RRR were used of 62.50% (95% CI: 9.79-84.41) and NNT of 7. The sample power was 64.40%.

**Table 3:** Comparison of atrial fibrillation within 72 postoperative hours, in-hospital death, hospital length of stay after coronary artery bypass grafting, number of grafts and use of cardiopulmonary bypass between patients with CVP control ≤ 15 cmH_2_O (G15) and CVP control ≤ 20 cmH_2_O (G20)

Variables	G15 (n = 70)	G20 (n = 70)	p
Atrial fibrillation, n (%)	6 (8.57)	16 (22.86)	0.03*
In-hospital death, n (%)	4 (5.71)	8 (11.42)	0.07*
Hospital length of stay, mean, days (CV)	7.14 (0.70)	8.21 (0.68)	0.36^†^
Grafts, median	3	2	0.22 ^†^
Cardiopulmonary bypass, n (%)	47 (67.10)	39 (55.70)	0.22*

(*) Fisher exact test (two-tailed);

(†) Mann-Whitney test (two-tailed); CVP: central venous pressure; CV: coefficient of variation.

In G15, there were 4 in-hospital deaths, 2 due to pulmonary sepsis and 2 due to stroke. In G20, there were 8 in-hospital deaths, 5 due to stroke, 2 due to pulmonary sepsis and 1 due to urinary sepsis. Mortality showed no statistical difference (p = 0.07). In addition, there was no difference regarding the post-CABG hospital length of stay (days), number of grafts, and use of CPB.

In the group of patients who developed AF, the following variables were analyzed: mortality, hospital length of stay, number of grafts, and use of CPB (Table 4). Age differed statistically between the groups (p = 0.04), with mean of 65.68 years among patients who developed AF, and of 60.73 among those who did not develop AF. The hospital length of stay was significant (p = 0.0012) among patients who developed AF, with mean of 10.22 days (CV = 0.70). The other variables were statistically similar.

**Table 4:** Comparison of age, mortality, hospital length of stay, number of grafts and use of cardiopulmonary bypass between patients who developed atrial fibrillation (AF) and those who did not

Variables	With AF (n = 22; 15.17%)	Without AF (n = 118; 84.29%)	p
Age, mean (CV)	65.68 (0.15)	60.73 (0.16)	0.04
In-hospital death, n (%)	2 (5.71%)	10 (11.42%)	1.00*
Hospital length of stay, mean, days (CV)	10.22 (0.70)	7.20 (0.67)	0.001^†^
Grafts, median	2	2	0.69^†^
Cardiopulmonary bypass, n (%)	12 (54.55)	74 (62.71)	0.48*

(*) Fisher exact test (two-tailed);

(†) Mann-Whitney test (two-tailed); CV: coefficient of variation.

## Discussion

This study compared the AF incidence in the first 72 post-CABG hours using two CVP control strategies, based on intention to treat, that is, the use of a diuretic was aimed at maintaining CVP below the cutoff points. Patients maintained with CVP ≤ 15 cmH_2_O in that period had lower AF incidence as compared to patients whose CVP was maintained ≤ 20 cmH_2_O (8.56% vs. 22.86%; p = 0.03). Measures of effect size were relevant: ARR of group G15 was 12.12%, equivalent to a NNT of 7, that is, 1 in every 7 patients benefited from CVP control ≤ 15 cmH_2_O after CABG, suggesting that maintaining CVP under control can be effective in reducing AF incidence. The AF incidences in each group and in the whole sample (15.71%) are similar to those reported in the literature (5-40%).^4^ The CVP was measured with a monitor in mm Hg and in water column; we chose to use the water column measure because some patients had only 24 hours of mmHg monitoring. For patients stable after 24 hours, it is routine procedure to end invasive blood pressure monitoring, CVP being controlled only by use of water column.

Atrial fibrillation in the PO period increases the risk for ischemic stroke, ventricular tachycardia, ventricular fibrillation, hypotension and heart failure.^3-7^ Post-CABG AF is associated with increased hospital length of stay after surgery and in-hospital mortality.^6^ Sobral et al.^9^ have reported a longer hospital length of stay of patients with AF (mean of 16.4 days; p = 0.004); however, they have not established whether AF was the cause of prolonged hospitalization or an indicator of the severity of more critically-ill patients. In addition, they have reported a peak incidence of 2.6 days (median, 2 days). Da Silva et al.^10^ have shown a mean hospital length of stay of patients who developed AF after cardiac surgery of 16.9 days (p = 0.001). In their sample, the hospital length of stay was significantly longer among patients with AF (p = 0.0012). They have not assessed the peak incidence in relation to that time, but the period studied (72 hours) is in accordance with the predicted time in the literature for higher AF incidence.^5,9,10^ The mortality rate reported by Sobral et al.^9^ 1 year after surgery was 4.7% (n = 109; p = 0.001) for patients who developed AF, with a 30-day rehospitalization rate of 7.6% (n = 168; p = 0.004) and an 1-year rehospitalization rate of 18.7% (n = 417; p = 0.004). In our sample, in-hospital mortality and hospital length of stay were assessed, and were slightly higher in G20, but with no statistical difference.

Knowing the risk factors for post-CABG AF is highly important. It enables the use of prophylactic measures, aimed at reducing the incidence of AF, as well as of its complications.^11,12^

Some risk factors for AF have been demonstrated. Age over 65 years^9,10^ has been reported as one of the most important risk factors.^7-12^ Age is associated with myocardial structural changes due to degenerative processes (fibrosis and dilatation), which lead to lack of an effective refractory period, dispersion of atrial refractoriness and abnormal conduction and automaticity.^4-7^ In addition, advanced age is related to increased in-hospital mortality.^9^ In our study, mean age was 60 years in G15, and 63 years in G20, with no statistical difference (p = 0.07). Age showed significance when analyzing patients who developed AF as compared to those who did not (p = 0.04). The mean age of those who developed AF was 66 years, similarly to that reported in the literature.^9,10^

Diabetes mellitus leads to metabolic changes, such as increased oxidative stress, elevated levels of free fatty acids, and chronic tissue inflammation. Such alterations result in changes in atrial structure and electrical conduction, contributing to AF development in the PO period.^5,13^ In our sample, the prevalence of diabetes mellitus was similar in both groups (p = 0.07).

Peripheral arterial occlusive disease associates with the severity of the patients' clinical profile and comorbidities that predispose to the appearance of AF after cardiac surgery.^9,14^ El-Chami et al.^14^ have identified peripheral arterial disease as a risk factor for AF, and considered it an independent predictor of mortality. In our study, the peripheral arterial occlusive disease prevalence did not statistically differ between the groups.

Other conditions associated with the development of post-CABG AF are COPD,^9,12,15^ chronic renal failure,^9,12^ previous AMI,^10,12^ and left ventricular dysfunction.^16,17^ The prevalence of those risk factors was similar in both groups.

The number of grafts performed during CABG and the use of CPB have been identified as risk factors,^7^ but with some disagreement between different authors.^9,10,14-19^ The CPB is an invasive technique related to atrial ischemia and inflammatory response in the PO period of cardiac surgeries.^7,20^ The groups studied showed no statistical difference regarding those parameters.

Some strategies for post-CABG AF prevention have been developed, especially those related to pharmacological prophylaxis. Beta-blockers are the drugs of choice, because they significantly reduce the AF incidence, being related to lower PO morbidity and mortality.^4,6,7,21^ They belong to the most studied and used drug class, especially because of the control they have on the increased sympathetic tone in patients submitted to cardiac surgery.^4,6^ Beta-blockers are indicated for all patients undergoing CABG, except in the presence of contraindications.^4,7,21^ Prophylaxis with amiodarone and intravenous magnesium is recommended when beta-blockers are contraindicated.^22^ In older studies, the use of statins in the preoperative and PO periods was considered relevant to post-CABG AF prevention. Those drugs act by reducing the inflammation of patients with coronary arterial disease. A meta-analysis by Zheng et al.^23^ has shown that statin therapy significantly reduced the AF incidence and hospital length of stay. Bockeria et al.^24^ have shown that patients receiving statin before CABG had higher benefits in preventing early AF than those who did not. However, the literature is still controversial. In a recent meta-analysis,^25^ the authors have concluded that PO statin therapy does not prevent AF in patients undergoing elective cardiac surgery. At our service, the use of beta-blockers and statins is maintained in the preoperative period and reintroduced with diet usually on the first PO day. If the patient is on dobutamine or noradrenaline, the beta-blocker is introduced after suspension of the vasoactive drug.

CVP is an important predictor of early mortality, independently of cardiac output and other variable clinical conditions, mainly in the first 6 PO hours, because of hemodynamic instability.^26^ Strict CVP control is aimed at measuring the pressure to which the atria are submitted, taking the intravascular volume into consideration. The result of the intravascular volume overload is hypertension, atrial dilatation and contraction reduction, because of stretching of cardiac muscle fibers. However, that can be reversed with diuretics.^26-28^ Kalus et al.^29^ have shown that hemodynamic control based on the administration of large amounts of fluids accounted for the increase in atrial pressure, and could trigger AF in the PO period of cardiothoracic surgery. They have observed that cardiothoracic surgery patients who developed AF had received approximately 1 liter of fluids more than those who did not develop that arrhythmia, and that difference was more significant on the second PO day (p = 0.04). The limiting factor of that study was the lack of documentation of the increase in atrial pressure and volume after surgery.

Intravascular volume overload causes abnormal dispersion of atrial refractory period, because of the increase in atrial volume and pressure. Thus, the atrium becomes vulnerable to the development of AF.^11,15,30,31^ Hwang et al.^31^ have suggested that intravascular volume is an important parameter, as are arterial gas analysis, hemoglobin and serum potassium, which should be assessed when there is post-CABG AF, because they can clarify the reversible causes of that arrhythmia. In addition, Silva et al.^10^ have shown that excessive fluid balance in the first 24 PO hours is a risk factor for post-CABG AF. Those authors have reported pulmonary congestion as a trigger for that arrhythmia. Koletsis et al.^15^ have reported an association between positive fluid balance, reflecting an excessive intravascular volume, and occurrence of post-CABG AF. In addition, the positive fluid balance has been accounted for the increase in left atrial pressure and pulmonary congestion, leading to hypoxia.

The increased left atrial volume identified in the preoperative period predisposes to the development of AF after cardiac surgery. Wang et al.^32^ have shown that the left atrial expansion index was associated with in-hospital mortality and post-CABG AF, being an independent risk factor. Osranek et al.^16^ have reported that an increase over 32 mL/m^2^ in left ventricular volume increases by 5 times the risk for AF, as a factor independent of age and other surgical parameters. Patel et al.^33^ have found that for every 5-mm increase in the left atrium, on the echocardiogram, the risk for AF increases by 39%. Sanfilippo et al.^30^ have concluded that maintaining sinus rhythm prevents the progression of left atrial hypertrophy and its adverse effects. Maceira et al.^34^ have studied the right atrial dimensions by use of magnetic resonance imaging. The best independent indicators of increased atrial volume were area greater than 16 cm^2^/m^2^ and longitudinal diameter greater than 3.5 cm/m^2^. The present study did not assess left atrial volume in the preoperative period, because not all patients underwent echocardiography before surgery.

In our study, CVP maintained ≤ 15 cmH_2_O proved to be important to prevent post-CABG AF, as compared to CVP ≤ 20 cmH_2_O. The statistical power of the sample was satisfactory (66.70%); however, further studies, with larger samples, are required to validate that approach as a prophylactic measure against AF in the PO period of cardiac surgery. Our data suggest that stricter CVP control is advantageous to prevent excessive volume overloads. Neither mortality after discharge, nor the appearance of AF after that have been assessed. Another study^35^ has shown that strict CVP control can prevent post-CABG AF as compared to no CVP control. Our study strategy proved beneficial (NNT = 7) in this relatively small sample (140 patients) in a single-center study.

Multicenter studies with a larger sample that can assess not only AF incidence, but also mortality and the increase in costs with longer hospital length of stay, are required to better assess this type of treatment. It is worth noting that the use of diuretics caused no harm to the patients, there was increase in neither renal failure nor hemodynamic instability, in addition to being a very low cost strategy. Thus, CVP control can serve as a complementary method in post-CABG AF prophylaxis.

## Conclusion

The CVP control maintained ≤ 15 cmH_2_O, in the first 48-72 PO hours, can reduce the incidence of post-CABG AF. One in every seven patients benefits from that strategy.
